# Boost your brain: a simple 100% normobaric oxygen treatment improves human motor learning processes

**DOI:** 10.3389/fnins.2023.1175649

**Published:** 2023-07-11

**Authors:** Zheng Wang, Guillaume Spielmann, Neil Johannsen, Frank Greenway, Brian A. Irving, Marc Dalecki

**Affiliations:** ^1^School of Kinesiology, Louisiana State University, Baton Rouge, LA, United States; ^2^Pennington Biomedical Research Center, Louisiana State University, Baton Rouge, LA, United States

**Keywords:** oxygen treatment (OT), sensorimotor system, visuomotor adaptation, motor learning, normobaric

## Abstract

**Introduction:**

Human motor learning processes are a fundamental part of our daily lives and can be adversely affected by neurologic conditions. Motor learning largely depends on successfully integrating cognitive and motor-related sensory information, and a simple, easily accessible treatment that could enhance such processes would be exciting and clinically impactful. Normobaric 100% oxygen treatment (NbOxTr) is often used as a first-line intervention to improve survival rates of brain cells in neurological trauma, and recent work indicates that improvements in elements crucial for cognitive-motor-related functions can occur during NbOxTr. However, whether NbOxTr can enhance the motor learning processes of healthy human brains is unknown. Here, we investigated whether a brief NbOxTr administered via nasal cannula improves motor learning processes during a visuomotor adaptation task where participants adapt to a visual distortion between visual feedback and hand movements.

**Methods:**

40 healthy young adults (*M* = 21 years) were randomly assigned to a NbOxTr (*N* = 20; 100% oxygen) or air (*N* = 20; regular air) group and went through four typical visuomotor adaptation phases (Baseline, Adaptation, After-Effect, Refresher). Gas treatment (flow rate 5 L/min) was only administered during the Adaptation phase of the visuomotor experiment, in both groups.

**Results:**

The NbOxTr provided during the Adaptation phase led to significantly faster and about 30% improved learning (*p* < 0.05). Notably, these motor learning improvements consolidated into the subsequent experiment phases, i.e., after the gas treatment was terminated (*p* < 0.05).

**Discussion:**

We conclude that this simple and brief NbOxTr dramatically improved fundamental human motor learning processes and may provide promising potential for neurorehabilitation and skill-learning approaches. Further studies should investigate whether similar improvements exist in elderly and neurologically impaired individuals, other motor learning tasks, and also long-lasting effects.

## 1. Introduction

A crucial part of our ability to manage daily tasks is learning from past experiences and practices to develop and improve non-inherited skills such as walking, reaching for a cup of coffee, or eating food with different tableware. Developing such skills is driven by human motor learning (Krakauer et al., 2019). However, the criticality of skill learning and retention in our daily lives is often not seen until learning capacity gets eclipsed or people need to re-learn daily functional movements due to aging or neurological diseases. For example, re-learning daily activities is a critical component of rehabilitation programs for stroke patients (Krakauer, 2006; Maier et al., 2019). Therefore, a large body of research has been conducted in the past decades to study fundamental and applied characteristics of human motor learning processes in healthy populations and individuals with neurological conditions. Research has shown that the human brain can learn and re-learn motor tasks (Ungerleider et al., 2002; Ostry et al., 2010). These learning processes are strongly related to the neuroplasticity of the brain’s neuronal system that strengthens its connections based on experience and usage levels (Dayan and Cohen, 2011) and also weakens with aging or as a result of pathological status and neurological impairments (Bock and Girgenrath, 2006). Therefore, any cost-efficient and easily accessible intervention treatment that could improve the capability of brain processes during learning or re-learning complex motor skills could be highly relevant not only for young adults but especially for aging individuals and clinical populations.

An essential factor for brain performance is efficient energy transfer, which depends on oxygen supply. Although the brain represents only 2% of the body mass, the brain consumes 20–30% of the body’s energy at rest (Lennie, 2003). Estimation models suggest that the optimal fraction of active neurons is about 1–4% (Attwell and Laughlin, 2001). Researchers argued that this limitation on energy supply from oxidative processes could be a rate limiter for the brain’s capacity to undertake complex tasks that require a larger amount of neuronal activity, which could be a potential limiting factor for brain function and plasticity (Scholey et al., 1999; D’haeseleer et al., 2011; Ota et al., 2013). In addition, reduced cortical blood and cerebral oxygen supply typically seen with aging, neurological conditions, or brain injuries likely further exacerbate such limitations (Edwards and Hart, 1974; Davis et al., 1983; Hadanny et al., 2020).

Further evidence for the essential role of oxygen is supported by studies with altered oxygen levels, where brain performance is enhanced in the presence of excess oxygen and reduced under hypoxic conditions (Scholey et al., 1999; Malle et al., 2013). Besides the well-known positive effects of higher levels of oxygenation on neuronal survival rates during neurological disease (Kelestemur et al., 2020), recent data suggest that a 100% normal baric oxygen treatment (NbOxTr) positively affects cognitive-motor-related functions, including improving reaction time and working memory (Chung et al., 2007; Choi et al., 2013; Malle et al., 2013). Such functions are crucial for motor learning processes (Thach, 1998; Sherwood and Lee, 2003), especially in the early phases of motor learning (Fitts, 1964). In addition, impaired performance was seen with insufficient oxygen supply when performing simple and complex psychomotor tasks (Bouquet et al., 1999). In sum, there is preceding evidence for the assumption that a lack of oxygen or an increased level of oxygen supply can affect brain function.

Visuomotor adaptation is the brain’s ability to adapt movements based on the information gained from visual feedback and the motor output to perturbations or distortions between vision and action (Krakauer et al., 2019). It is highly related to brain plasticity and crucially important for daily life since most upper-limb motor functions are linked to coordinating eye-hand movements and to adapt actions accordingly while practicing them (Della-Maggiore et al., 2015). Visuomotor adaptation requires the transformation, integration, modification, and storage of visuospatial and kinesthetic information to reduce aiming errors, for example when a sudden distortion between visual feedback and motor output is installed (Bastian, 2008; Della-Maggiore et al., 2015). Humans correct these errors by adjusting their movements accordingly, trial by trial. They often adapt more and faster during the early learning phase when the error size is larger, with the correction amplitude being proportional to the error magnitude (Anguera et al., 2010; Vachon et al., 2020). Oppositional directional errors, often called “After-Effects,” usually occur as a result of the learning process when the perturbation between action and perception is removed and usually decrease significantly within a short amount of time (Huang et al., 2011; Hadjiosif and Smith, 2013; Kitago et al., 2013). Notably, the fast disappearance of these After-Effects does not translate into the disappearance of the motor adaptation learned in the previous adaptation phase. When participants are exposed to the same distortion again in a “Refresher” phase, they usually start with a lower error magnitude which also declines faster compared to the initial adaptation phase (Hadjiosif and Smith, 2013). Not surprisingly, visuomotor adaptation abilities usually undergo notable changes with deteriorating brain performance levels as well (Contreras-Vidal and Buch, 2003; Corzani et al., 2019).

The motor learning processes of visuomotor adaptation critically depend on information processing and memory functions (Bock, 2005). Considering the sensitivity of these learning-related processes to circulating oxygen levels, it is likely that motor learning processes of visuomotor adaptation could be positively affected by higher levels of supplemented oxygen. Interestingly, to our knowledge, whether human motor learning processes can be improved by a simple and easy to administer NbOxTr is unknown. Therefore, the present study examined the effects of a NbOxTr on human motor learning processes while performing a visuomotor adaptation task. We hypothesized that increasing the oxygen supply may significantly facilitate motor learning processes during the visuomotor adaptation task, specifically during the early learning stages in the adaptation phase. We also hypothesized retention of these effects after the treatment had been removed in the subsequent experiment phases that follow the adaptation phase, more likely under task conditions similar to the one where the oxygen treatment has been provided.

## 2. Materials and methods

### 2.1. Participants

In total, 40 right-handed healthy young adult participants (10 males, 30 females; mean age: 21.8 years) were recruited for this study. Most of the participants were recruited from the undergraduate student population at Louisiana State University. Participants were randomly assigned to two groups: a group who received a treatment with 100% normobaric oxygen (group “NbOxTr,” *n* = 20, mean age: 21.65 ± 1.53 years, 5 males), or a group who received a treatment with medical air (group “AirTr,” *n* = 20, mean age: 21.95 ± 2.87 years, 5 males) during a specific phase of the visuomotor adaptation task. Handedness was assessed by the Edinburgh Handedness Inventory questionnaire. Participants who reported having experience participating in any visuomotor experiments were excluded from this study. The protocol of this study was approved by the Human Subjects Institutional Review Board at Louisiana State University (IRB #4341). *A priori* power analysis performed by G-power software (Version 3.1) revealed a sample size of at least 36 participants (power = 0.95, effect size = 0.25) for each variable if repeated measures of ANOVA was run. Typical visuomotor adaptation experiments comparing two groups using a similar design (i.e., a Baseline, Adaptation, After-Effect, and Refresher phase) included 20 participants in each group (Simon and Bock, 2017), such that we decided to include 40 participants in our study as well, with 20 participants in each group.

### 2.2. Equipment

**FIGURE 1:**
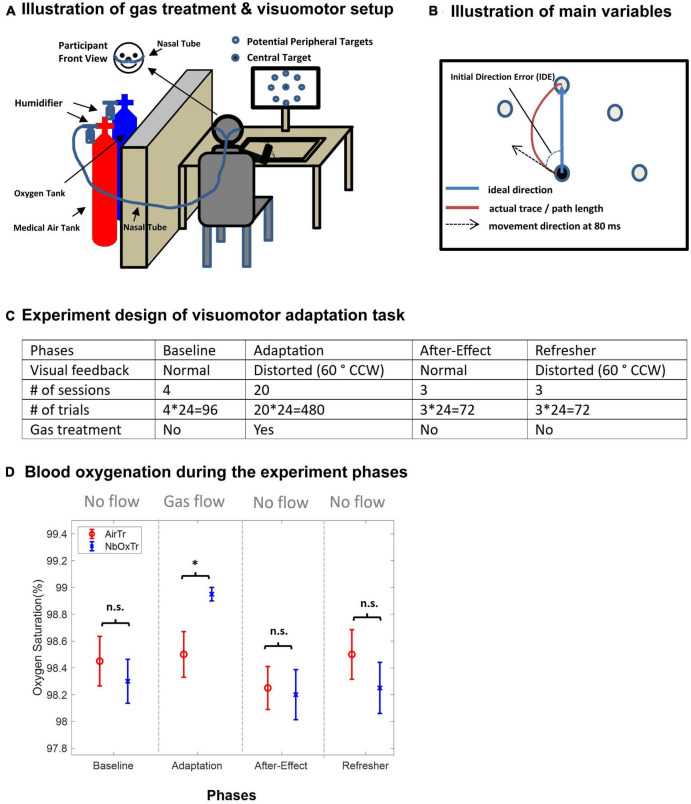
Normobaric 100% oxygen treatment (NbOxTr), motor learning setup and design and blood oxygenation during the experiment phases. **(A)** Schematic illustration of the oxygen treatment and the visuomotor adaptation task setup. **(B)** Illustration of the main movement variables (IDE, PL) of the motor learning task. **(C)** Experiment design of the visuomotor adaptation task. Note that a gas treatment was only administered during the Adaptation phase, medical air for AirTr group and 100% oxygen for NbOxTr group, After-Effect, and Refresher phases. There was a 3-min break between Baseline and Adaptation and another 3-min break between Adaptation and After-Effect. CCW, counter clock-wise. **(D)** Mean blood oxygenation for both groups, NbOxTr and AirTR, during the Baseline, Adaptation, After-Effect, and Refresher phases. Blood oxygenation was similar between both groups during the Baseline, After-Effect, and Refresher phases but was significantly higher in the NbOxTr group than in the AirTR group during the Adaptation phase; n.s., non-significant, *P < 0.05; error bars represent the standard error of the mean.

A graphical illustration of the experiment setup is provided in Figure 1A. Visuomotor data were collected via a horizontally placed digitalized tablet and a stylus pen (Wacom Intuos Pro Pen and Touch Tablet), which were connected to a Dell Inspiration desktop monitor (Dell U2417H 24′′) and adjusted to the height of participants’ eye level. During the experiment on the first day, participants were equipped with a nasal cannula (Salter 1600HF High Flow Nasal Cannula) connected to an oxygen regulator (Oxygen Gas Regulator, CGA-540, Single Stage, Brass, 4 to 80 psi) connected to an oxygen tank (Airgas Medical oxygen (100%) tank, size 200), or to a regular flow regulator (Regular Gas Regulator, Single Stage, Brass, 4 to 80 psi) connected to a tank with regular air (Airgas Regular Medical Air, 21% oxygen, size 200). The oxygen tank and the regular air tank were both secured in a separate cylinder stand and stored behind a wall outside participants’ sight. A standard bubble humidifier (Salter Labs 6-15 LPM High Flow 350cc Bubble Humidifier) was installed between the nasal canula and the flow regulator. An air measuring device (BW Honeywell Clip 2 Year O2 Single Gas Detector BWC2-X) was placed in the experiment room to monitor ambient air continuously, and a pulse oximeter with continuous blood oxygen saturation recording function (EMO-80, EMAY) was placed on the index finger of the left hand to record the oxygenated hemoglobin (HbO2) level of the participants during the experiment.

### 2.3. Experiment design

The experiment design of the visuomotor adaptation task is illustrated in Figure 1C. For the visuomotor adaptation (VMA) task, different conditions were presented by a program set up in Movalyzer (Neuro-Script LLC, Tempe, AZ). Participants were instructed to put the pen tip at the center of the central target and then move to the center of one of the eight peripheral targets, which were 45 degrees to each other and placed 7.5 cm from the central targets. Data of the pen tip position was sampled at 100 Hz. Targets were all solid black dot with 0.75 cm radius and would show up randomly. Peripheral targets would not appear until the pen tip was placed at the center of the central target and the central target would disappear as soon as the peripheral target appeared. There were four phases of the VMA task: 4 sessions of Baseline, 20 sessions of Adaptation, 3 sessions of After-Effect, and 3 sessions of Refresher. Each session contains 24 trials, with 3 trials to each of 8 possible peripheral targets. The cursor movement on the screen correctly represented the movement of stylus pen during the Baseline phase and After-Effect phase. While during Adaptation phase, the cursor movement was rotated 60-degree counter clock-wise during the Adaptation and Refresher phase, in another word, the visual feedback was distorted during these two phases. Participants were instructed to move as fast and as accurately as they can, and that the experiment complexity may increase or change during the experiment.

During the experiment, we provided a 100% normobaric oxygen treatment (5 L/min) via a nasal cannula only during the Adaptation phase of a visuomotor adaptation task. The control group received a similar treatment but with normobaric medical-grade air (AirTr) (i.e., regular air with 78% nitrogen, 21% oxygen, and 0.04% carbon dioxide). The Adaptation phase (trials with a 60-degree distortion between visual feedback and hand movement) started 3 min after the gas flow was turned on. After the Adaptation phase, the gas flow was turned off, and following a 3 min washout phase, the experiment continued with the After-Effect phase of the visuomotor task with normal visual feedback (i.e., similar to the Baseline) to investigate the savings of the adaptation to the distorted feedback. Finally, in a Refresher phase which did not include gas flow as well, the visual distortion was reinstated again to scrutinize whether potential improvements gained in the Adaptation phase with the oxygen treatment were consolidated further into the post-treatment period.

### 2.4. Procedures

The participants were first presented with the consent form and procedures were explained before continuing. After participants have given their informed consent to participate, they were asked to fill out a summary sheet about their demographic information, neurological disease history, and paper-pencil based assessments of sleep quality and handedness (Edinburgh Inventory) tests, with questions in a Likert-format. For further details, see Oldfield (1971), Hart and Staveland (1988). Finally, participants were equipped with the nasal cannula and pulse oximeter. A familiarization period containing 24 Baseline trials of the VMA task was used before the main experiment started. First, 4 Baseline sessions were performed with visual feedback correctly representing the pen position (“Baseline” phase), followed by 20 sessions with 60°clockwise rotated visual feedback (“Adaptation” phase), 3 sessions with correct visual feedback (“After-Effect” phase), and finally again 3 sessions with 60°clockwise rotated visual feedback (“Refresher” phase). Breaks were indicated by participants. In total, 30 sessions, with 720 trials (24 × 30) were performed for each participant throughout the experiment (see Figure 1C).

The gas treatment with either 100% oxygen for the 100%NbOxTr group or medical air with 21% oxygen for the AirTr group was delivered only during the 20 adaptation sessions in the adaptation phase. Participants started to receive air or oxygen at a flow rate of 5 L/min after they finished Baseline phase. The adaptation phase was then started 3 min after beginning of the gas delivery. The gas delivery was stopped when the Adaptation phase was completed, and the After-Effect phase started after a 3-min break. Stress level (NASA TLX) was assessed after the completion of the visuomotor adaptation task.

### 2.5. Data analysis

Data analysis of the visuomotor task was performed using custom-written MATLAB (MathWorks) code. Pen tip position data was filtered by a Butterworth 4th order dual pass filter with a cut-off frequency of 7 Hz. The onset and termination of the pen movement were identified by a fixed criterion, 5% of the peak velocity. Main variables were initial directional error (IDE,°) and path length (PL, mm) (see Figure 1B for illustration). Other variables of interest are reaction time (RT, ms), movement time (MT, ms) and absolute error (AE, mm). The IDE was defined as the movement angle (at 80 ms from onset) in relation to the straight line between the centers of the central and target circle. The PL was defined as the pen trajectory length from the onset to the termination of the movement. The MT was defined as the duration between the onset and termination of the whole pointing movement. The RT was defined as the duration from the appearance of peripheral targets to the onset of the movement. The AE was defined as the distance between movement termination spot and centers of peripheral targets. For each experiment phase, percentage differences between groups (NbOxTr, AirTr) was calculated as [(performance of NbOxTr-Performance of AirTr)/Performance of AirTr) × 100%].

Finger oximeter data was sampled by a standard pulse oximeter (EMO-80 Sleep Oxygen Monitor, EMAYCARE) at 1 Hz, and the average value was calculated for each session during the experiment for each participant.

A multivariate analysis of variance (MANOVA) was performed first for all the motion data, oximeter data and questionnaire data via STATISTICA, version 25. After a significant difference was identified, repeated-measures analysis of variance (ANOVA) was performed for all visuomotor data and two-sample *t*-test for oximeter and questionnaire data. An analysis with Sex as covariate did not reveal significant effects of the factor Sex (all *p* > 0.05), such that we tentatively suggest outcomes reported in our study were not confounded by Sex. All data were checked for normal distribution (Shapiro–Wilk’s test) and sphericity (Mauchly’s test) and Greenhouse-Geisser corrected in case of sphericity violations. Statistical significance levels were set to α < 0.05.

## 3. Results

The assessments to assess their subjective sleep quality and stress level revealed comparable perceived sleep quality before participating and similar stress levels after completion of the experiment task between both groups (all *p* > 0.05; for descriptive results and statistical details, see Supplementary Material IV). Notably, oxygen saturation (SaO2) levels were higher in the NbOxTr group than in the AirTr group only during the Adaptation test session when the gas treatment was provided (*p* < 0.05), and were not significantly different between groups in all other experiment sessions (all *p* > 0.05), see Figure 1D (for descriptive results and statistical details, see Supplementary Material III). Descriptive results and statistical details of the visuomotor adaptation experiment are summarized in Supplementary Materials I, II.

### 3.1. Crucial motor learning characteristics linked to movement planning were enhanced during the oxygen treatment and remained better afterward

The normobaric 100% oxygen treatment of 5 L/min administered via a nasal cannula significantly improved the speed and rate (+26%) of central characteristics of visuomotor adaptation (i.e., the initial direction of the movement stroke) when participants learned to adapt to a visual distortion between hand movement and visual feedback. Notably, this advanced learning effect of the NbOxTr group over the AirTr group was consolidated into the subsequent experiment phases without gas treatment when the visual distortion was removed (+36%) and later reinstated (+25%) again.

**FIGURE 2:**
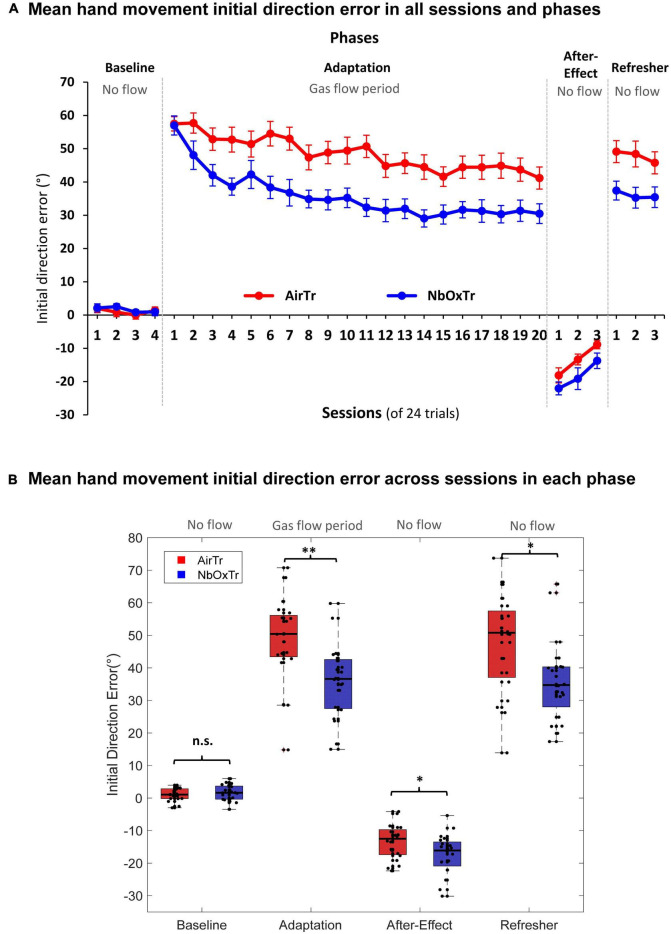
The oxygen treatment improves crucial motor learning characteristics linked to movement planning. **(A)** Mean IDE in each experiment phase and across sessions for both groups, NbOxTr and AirTR. **(B)** Mean IDE across each experiment phase (Baseline, Adaptation, After-Effect, Refresher) for both groups, NbOxTr and AirTR. n.s., non-significant, *P < 0.05, ^**^P < 0.01. Boxes represent the 25 to 75 percentile and whiskers represent 2.7 times of standard deviation. Black dots represent individual data points. Plots for Adaptation, After-Effect, and Refresher in panel **(B)** are based on normalized data subtracted from the mean Baseline for any given participant and session.

During the Baseline phase of the visuomotor adaptation task, no significant differences between groups were observed for the IDE, and no significant interaction between Group × Session either (all *p* > 0.05). This result suggests a similar IDE performance between the NbOxTr and AirTr groups during the Baseline phase with normal visual feedback and without gas treatment. However, these patterns changed notably in the next phase (i.e., the Adaptation phase) after the gas treatment (i.e., 5 L/min flow via nasal cannula) was turned on in both groups (NbOxTr, AirTr). Movement-planning related characteristics were superior in the NbOxTr compared to the AirTr during the Adaptation phase when participants learned to adapt to the distortion between the pen movement and the cursor feedback on the display (i.e., participants in the NbOxTr group had a smaller IDE, see Figure 2B). The IDE was 26% lower across the Adaptation phase in the NbOxTr than in the AirTr. Notably, there was a significant interaction between Group × Session (*p* < 0.01), suggesting that the Group effect was dependent on the factor Session. *Post hoc* analysis suggested that the adaptation to the visual distortion was significantly faster in the NbOxTr group than in the AirTr group at the early beginning of the Adaptation phase, specifically during the first four adaptation sessions. It seems that the NbOxTr group reduced their IDE by episode 2 to an amount the AirTr group reached by episode 8, in episode 3 to an amount the AirTr group reached in episode 15, and in episode 4 an amount the AirTr group reached by episode 20, respectively. Afterward, the learning pattern along the remaining sessions during the Adaptation phase with gas treatment and visual distortion looked rather similar between groups (see Figure 2A).

In detail, during the After-Effect phase of the visuomotor experiment, when the distortion between hand movement and visual feedback on the display was removed, and hand and cursor movement were aligned again (i.e., same conditions as during Baseline), there was a significant Group effect in the After-Effect phase found for the IDE (*p* < 0.05). The IDE was significantly larger in the NbOxTr group (+36.11%) than in the AirTr group (i.e., higher IDE into the negative direction) during the After-Effect phase after the removal of the 60-degree rotation (see Figure 2B), suggesting better “saving” of the adapted behavior in the NbOxTr group after the removal of the visual distortion. No significant interaction was observed between Group × Session (*p* > 0.05), suggesting a constantly larger IDE in the NbOxTr group across the After-Effect phase (see Figure 2A). Note that no gas treatment was provided during the After-Effect phase and participants breathed normally but were still wearing the nasal cannula, similar as in the Baseline phase. In the last phase, the Refresher, the visual distortion between pen movement and the cursor on the visual display was re-instated again, such that the visuomotor conditions were similar to the initial Adaptation phase with the same distortion. However, no gas treatment was administered in the Refresher phase. Notably, a significant group effect in the Refresher phase (*p* < 0.05) revealed a 24.6% lower IDE in the NbOxTr compared to the AirTr when participants re-adapted to what they learned during the previous Adaptation phase where they received the gas treatment (see Figure 2B). In the Refresher phase, no significant interaction was observed between Group × Session (*p* > 0.05), revealing that the Group effect was independent from sessions, suggesting a generally lower IDE in the NbOxTr group over the course of the Refresher phase (see Figure 2A).

### 3.2. Crucial motor learning characteristics linked to movement execution were enhanced during the oxygen treatment and remained better afterward

During the visuomotor adaptation experiment, motor learning-related characteristics linked to movement execution (i.e., movement straightness) significantly improved in the NbOxTr group (i.e., 11% shorter PL), in particular in the very early phase of the Adaptation phase. The movement smoothness advantage of the NbOxTr group over the AirTr group became smaller but remained present throughout the Adaptation phase while participants adapted to the visual distortion between cursor feedback and pen movement while also receiving the gas treatments. The shorter PL in the NbOxTr group was not consolidated into the following After-Effect phase when the gas treatment and the distortion between pen stroke and visual feedback were removed. However, advanced learning of motor execution related patterns in the NbOxTr group consolidated into the final Refresher period (i.e., an 11% shorter PL) when the visual distortion (i.e., a task similar to what participants adapted to in the Adaptation phase with gas treatment) was reinstated again.

**FIGURE 3:**
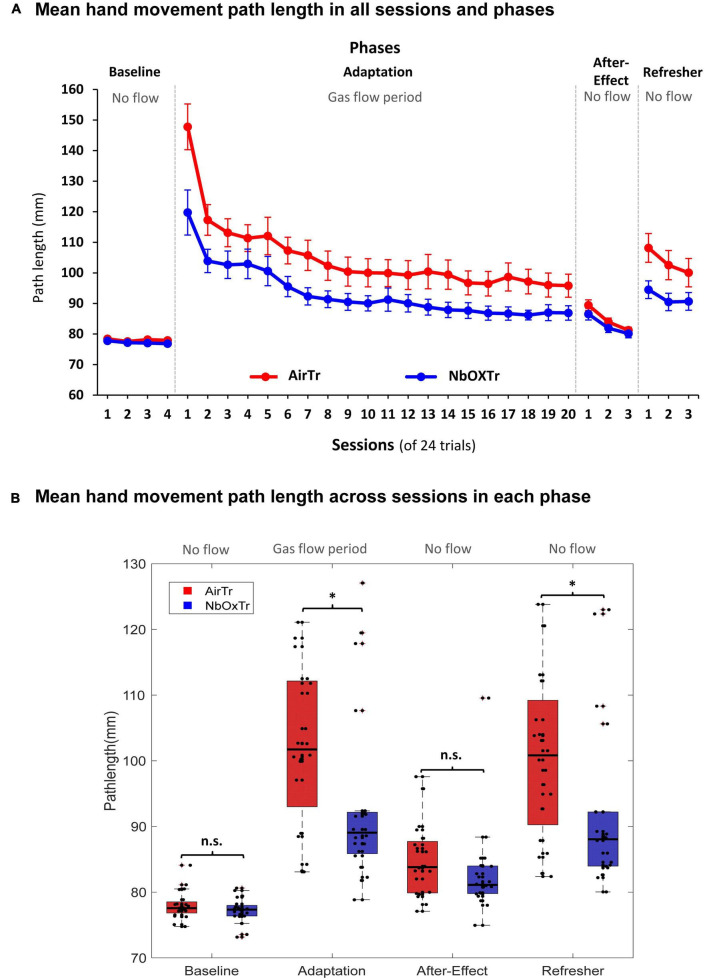
The oxygen treatment improves crucial motor learning characteristics linked to movement execution. **(A)** Mean PL in each experiment phase and across sessions for both groups. **(B)** Mean PL across each experiment phase (Baseline, Adaptation, After-Effect, Refresher) for both groups. n.s., non-significant, *P < 0.05. Boxes represent the 25–75 percentile and whiskers represent 2.7 times of standard deviation. Black dots represent individual data points.

In detail, in the Baseline phase of the visuomotor adaptation experiment, the PL of the pen stroke (i.e., of the hand movement) was similar between the NbOxTr and AirTr group (see Figure 3B), and there was also no significant interaction between Group × Session (cf., Figure 3A) (all *p* > 0.05). This suggests a similar movement execution performance regarding the straightness of the movement from the center to the peripheral target in both groups during the initial phase of the experiment with normal visual feedback and no gas treatment. This pattern then changed notably in the Adaptation phase after the gas treatment was turned on and the installation of the visual distortion between the cursor on the display and the pen stroke on the horizontal tablet. There was a significant Group effect in the Adaptation phase (*p* < 0.05), suggesting an overall shorter PL in the NbOxTr group (−10.90%) than in the AirTr group (see Figure 3B). A significant interaction between Group × Session revealed that this group effect was dependent on the factor session (*p* < 0.01). *Post-hoc* test analysis revealed that the first session largely drove this effect during the Adaptation phase, where the PL was drastically shorter in the NbOxTr (−18.97%) than in the AirTr. The difference between both groups became notably smaller by the second session of the Adaptation phase and remained consistent then throughout the rest of the Adaptation phase (see Figure 3A).

In the After-Effect phase, there was no significant group effect and also no significant interaction between Group × Session (all *p* > 0.05), suggesting similar motor execution patterns regarding the straightness of the movement stroke from the center to the peripheral target in the phase after the gas treatment, and the visual distortion were both removed. However, again, it was a different picture in the Refresher phase. Here, a significant Group effect was found when the visual distortion from the Adaptation phase was again reinstated in the Refresher phase, revealing a significantly shorter PL in the NbOxTr (−11.30%) than in the AirTr (*p* < 0.05). A significant interaction between Group × Session revealed that the Group effect was dependent on session (*p* < 0.05). *Post-hoc* test analysis showed that the effect was more pronounced during the first two sessions but could be consolidated into the last session of the Refresher phase (see Figure 3A).

Reaction time (RT, ms), movement time (MT, ms) and absolute error (AE, mm) were also analyzed in each phase and no significant differences between groups were identified (all *p* > 0.05). Further details for RT, MT, and AE are provided in Supplementary Materials I, II, V.

## 4. Discussion

Here we demonstrate that a normobaric 100% oxygen treatment (NbOxTr), administered via a nasal cannula, can improve immediate motor learning processes in healthy young adults. These findings suggest that a simple and easy-to-administer NbOxTr can improve human motor learning processes in healthy young adults. Notably, the majority of these improvements were consolidated after the gas treatment was terminated. Although we observed consistent motor learning improvements, further studies should elucidate to which extent such improvements can be consolidated in the long-term, whether they occur in other motor learning tasks, as well as in older or neurological populations, and the mechanisms behind such changes. Nevertheless, this study provides the first evidence that human motor learning processes can be significantly improved by providing normobaric oxygen via a nasal cannula during the acquisition period of skill learning. A potential explanation for the substantial motor learning improvements found in a typical visuomotor adaptation task during and after an NbOxTr may be related to the nature of the task and the susceptibility of particular brain functions to normobaric oxygen therapy. Further details will be discussed in the subsequent sections.

Notably, the largest skill acquisition benefit of the NbOxTr group, when compared to the AirTr group, occurred seemingly in the early period of the Adaptation phase within the first few sessions (see Figures 2A, 3A). In Fitts and Posners model of motor learning, the first stage of learning was named as cognitive stage (Fitts, 1964). In this phase, more spatial and temporal movement components are learned, and more neural networks are activated than in later phase, such as cortico-striatal loops and cortico-cerebellar loops (Hardwick et al., 2013). As a result, cognitive functions or strategical control were reported to contribute more in the early phase than in later phases of motor learning (Simon and Bock, 2017). Furthermore, oxygen treatment has been reported to have positive effects on multiple cognitive functions, such as memory function (Chung et al., 2007). Since spatial memory is a significant contributing factor to the visuomotor adaptation process (Anguera et al., 2010), this could be an important mechanism by which NbOxTr may improve visuomotor adaptation performance.

Functional Magnetic resonance imaging (fMRI) studies are consistent with this hypothesis. Research has shown that some cerebellar functions act as an error-detector during visuomotor adaptation, which compares expected sensory input of movement with actual sensory input (Miall and King, 2008). As discussed above, the reduction of the IDE is more evident in the early phase than later phase during the adaptation process, and the cerebellum was also seen to be activated more during the early phase (Ungerleider et al., 2002). Thus, this brain area may require more energy transfer during the early phase than later. Therefore, providing more oxygen might have boosted learning processes during this early period of the adaptation process, potentially by supporting enhanced glucose oxidation (Shulman et al., 2001, 2002). Further fMRI studies have also shown a strong connection between spatial-related areas in the cerebellum and cognitive areas in the frontal lobe during both cognitive and motor tasks (Diamond, 2002), providing additional support for our hypotheses. Furthermore, a fast reduction of the IDE during the early adaptation process occurred only in the NbOxTr group (i.e., from session 1 to 2) but not in the AirTr group (i.e., no drop from session 1 to 2), a pattern distinct from PL (a similar drop from session 1 to 2 in both groups). This finding seems to further underline that the oxygen treatment may have improved particularly cognitive-related visuomotor resources in the early phase of adaptation, as solely the variable especially sensitive to cognitive resources (i.e., IDE) showed treatment sensitivity and distinct patterns between group.

Another visuomotor adaptation strategy, the proprioceptive recalibration/spatial realignment, was described as the slow learning component and relies on the discrepancy between expected and actual sensory input (Della-Maggiore et al., 2015). Little research has been conducted on the direct impact of oxygen treatment on proprioception. However, some research reported already that the tonic tendon vibration reflex amplitude, which is a way to assess the proprioception contribution to motor task, was reduced under a normobaric hypoxia environment (Delliaux and Jammes, 2006; Debenham et al., 2021). Proprioceptive recalibration plays an important role in visuomotor adaptation tasks (Henriques and Cressman, 2012), which was potentially positively affected by the oxygen treatment as well. This could be one of the reasons why participants who received the oxygen treatment were able to maintain the earlier learning improvements also into the later phases of the adaptation sessions but also into subsequent experiment phases where no gas treatment was given that proved short-term consolidation. Indeed, performance variables such as the IDE and the PL in the Refresher phase are heavily linked to sensory recalibration processes (Cressman and Henriques, 2009; Krakauer and Mazzoni, 2011; Krakauer et al., 2019).

When comparing the main variables of interest regarding movement planning (i.e., IDE) and movement execution (i.e., PL) during the adaptation period, it seems that the movement planning related characteristics showed a stronger response to the oxygen treatment (i.e., 26% smaller IDE in the NbOxTr group than AirTr group) than the movement execution related patterns (i.e., 11% shorter PL in the NbOxTr group than AirTr group). This difference between the behavioral outcomes of planning (IDE) and execution (PL) related movement patterns is not surprising when considering that essential characteristics related to motor planning can be linked to cognitively driven elements such as strategic control processes and working memory (Bock, 2005; Bo et al., 2009). These cognitive functions are critical for motor learning (Sherwood and Lee, 2003), have been reported to improve with supplemental oxygen (Choi et al., 2013; Malle et al., 2013), and are highly dependent on oxidative metabolism (Scholey et al., 1999; Shulman et al., 2001).

This finding is also in line with the observation found in the present work that superior learning progresses in the oxygen treatment group mainly appeared in the early phase of the adaptation process during the Adaptation phase, a phase linked to the “cognitive stage of learning” in Fitt’s well-known learning phase model (Fitts, 1964) (see above). Interestingly, similar patterns have been observed also in the following phases without gas treatment (i.e., After-Effect and Refresher), where consolidated learning advantages in the oxygen group were consistently larger for motor planning (i.e., IDE) than for movement execution (i.e., PL) related characteristics. This is potentially a result of the benefits acquired in the early adaptation period during the cognitive stage of learning in the Adaptation phase. It also seems that other movement planning and execution related variables (i.e., RT, MT, and AE) were not affected by the NbOxTr, suggesting that those characteristics were not sensitive to the oxygen treatment administered during a typical visuomotor adaptation task. Further research could scrutinize whether the same pattern of findings is true in a learning task that has fewer motor but more cognitive components, such as a sequence learning task.

Importantly, both study groups had comparable perceived values of sleep and stress levels measured by questionnaires before they started the visuomotor experiment, suggesting that those characteristics would unlikely be responsible for the learning differences that occurred throughout the visuomotor experiment phases between groups. Furthermore, the performed oxygen treatment increased hemoglobin oxygen saturation levels, as measured using standard pulse oximetery (fingertip on the left index finger) in the NbOxTr group during the gas treatment period, and blood oxygenation levels were on a similar level between both groups in all other experiment phases (i.e., Baseline, After-Effect, and Refresher phase).

We can only speculate as to why the oxygen treatment improved motor learning processes, as our experiment design focused on behavioral data of the movement in concert with measurements of the blood oxygen level measured at the fingertip. We showed that blood oxygen saturation measured at the standard pulse oximetry was significantly higher in the NbOxTr group compared to AirTr only during the Adaptation phase, while the blood oxygen saturation was similar between both groups in all other experiment phases (Baseline, After-Effect, Refresher). However, there are well-known effects on oxygen levels in the bloodstream and brain with similar 100% oxygen treatments and comparable flow rates (e.g., 5 l/min), for example, an increased arterial partial pressure of oxygen (PaO2) (Scheeren et al., 2018). An increased PaO2 is a driving force of oxygen diffusion into the tissue, including elevating blood oxygenation levels in the brain (Chung et al., 2007). Our oxygen treatment has likely elevated the arterial oxygen tension and facilitated the transportation of oxygen from the arterial vessels to the brain tissue. fMRI studies also reported a universal phenomenon named the “initial dip” (Xu et al., 2012), which showed local oxygenation decrease prior to the responsively increased blood flow change to neural activities. We postulate that the increased PaO2 induced by our oxygen treatment may have helped during this gap period and maintained tissue oxygenation to some extent. However, this assumption needs to be confirmed by future fMRI or NIRS studies. Our laboratory is currently investigating these matters. Interestingly, cerebral blood flow (CBF) seems to be mostly unaffected by hyperoxia and an increased PaO2 (Xu et al., 2012), suggesting an increased blood flow may have played a rather minor role for the proposed facilitated transportation of oxygen to the brain tissue.

Our study has limitations. As mentioned previously, our simplified approach was limited to behavioral aspects and only included *in vivo* measurements of oxygen levels conducted at the fingertip and not at the brain itself. Investigating the oxygenation levels of the brain, for example, using Near Infrared Spectroscopy, would provide a greater understanding of the physiological mechanisms behind the improvements in learning. In the current study, both groups showed poor learning during the Adaptation phase, as indicated by the limited reduction in IDE (which was still ∼ 40° in session 20, see Figure 2A). This could be due to several factors. Firstly, using eight peripheral targets in the experiment may have weakened the learning process compared to using fewer targets, as previous research suggests (Krakauer et al., 2000). Additionally, the visibility of the reaching hand during the experiment might have contributed as well. In tasks involving visuomotor adaptation, the amount of visual feedback from the reaching hand has been found to influence the learning process. In similar tasks where the hand was covered by blinds, participants commonly achieved a greater reduction in IDE [e.g., an IDE < 20° (Simon and Bock, 2017)]. Reduced visual feedback prompts participants to rely more on proprioception, leading to improved visuomotor remapping and the development of the internal model associated with movement planning (Shabbott and Sainburg, 2010). However, in our study, we kept the right hand uncovered to maintain ecological validity. Future studies could explore whether altering the visibility of the hand and relying more on proprioception would result in stronger or weaker treatment effects as reported in our study.

In addition, researchers debate whether visuomotor adaptation can serve as a solid model for motor skill learning (Fleury et al., 2019). Thus, follow-up studies using a different motor learning task would shed more light on task-specific aspects of learning. This study investigated the effects of oxygen therapy on learning in young adults. Future research should investigate whether older adults, and clinical neurological patients, have greater responses for learning to oxygen therapy over longer periods of time, since they may have impaired oxygen delivery function. Last, measuring long-term consolidation of improved learning effects would be important, especially as consolidation beyond the treatment day would be crucially relevant for clinical applications (Krakauer, 2006). In future studies, the effectiveness of this treatment could be investigated in neurological populations (e.g., stroke patients) and in acute versus chronic treatment scenarios as well.

In summary, this study was the first to show that a simple and brief normobaric 100% oxygen treatment administered via nasal cannula (NbOxTr) can substantially improve motor learning processes compared to regular air treatment in healthy young adult humans. The NbOxTr lead to substantially faster and about 30% better learning during a typical visuomotor adaptation task period where participants adapted to a visual distortion between visual feedback and hand movements. We also demonstrate that the participants were able to consolidate these improvements after the termination of the oxygen treatment, at least in the short-term, as shown in the After-Effect and Refresher phases of the visuomotor adaptation experiment. This NbOxTr technique may have promising potential for neurorehabilitation and skill learning approaches. Future studies should scrutinize long-term consolidation and the physiological mechanisms behind the improved learning processes, and whether such improvements occur in elderly individuals, neurological populations and in other motor learning tasks as well.

## Data availability statement

The original contributions presented in this study are included in this article/Supplementary material, further inquiries can be directed to the corresponding author. The full data set is provided in Supplementary file V.

## Ethics statement

The studies involving human participants were reviewed and approved by the Human Subjects Institutional Review Board at Louisiana State University (IRB #4341). The patients/participants provided their written informed consent to participate in this study.

## Author contributions

ZW: conceptualization, methodology, data collection, analyzing, and writing of the original manuscript draft. GS, NJ, and BI: methodology, writing — reviewing, and editing. FG: methodology, data collection, writing — reviewing, and editing. MD: conceptualization, methodology, data analyzing, writing — reviewing, and editing. All authors contributed to the article and approved the submitted version.
