# Supplementary material for: Boost your brain: a simple 100% normobaric oxygen treatment improves human motor learning processes
Source: Front Neurosci. 2023 Jul 11;17:1175649. doi: 10.3389/fnins.2023.1175649 (PMC10366362; doi:10.3389/fnins.2023.1175649)
Supplement: Supplementary file 1 [file Table_1.DOCX]

**Suppl. file I:** Visuomotor adaptation task mean descriptive results of the repeated-measures mixed ANOVA’s for Baseline, Adaptation, After-Effect, and Refresher phases for Group (NbOxTr, AirTr) and Session (Baseline 1-4; Adaptation 1-20; After-Effect & Refresher 1-3) for all dependent variables (IDE, PL, RT, MT, AE). Note, gas treatment was only provided during the Adaptation phase. Abbreviations: NbOxTr = 100% normobaric oxygen treatment, AirTr = medical air treatment; IDE = Initial Direction Error, PL = Path length, RT = Reaction time, MT = Movement time, AE = Absolute endpoint error.

| **Baseline Phase** | | | | | | **After-Effect Phase** | | | | | **Refresher Phase** | | | | |
| --- | --- | --- | --- | --- | --- | --- | --- | --- | --- | --- | --- | --- | --- | --- | --- |
| **Variable** | **Session** | **NbOxTr** | | **AirTr** | | **Session** | **NbOxTr** | | **AirTr** | | **Session** | **NbOxTr** | | **AirTr** | |
|  |  | **Mean** | **SD** | **Mean** | **SD** |  | **Mean** | **SD** | **Mean** | **SD** |  | **Mean** | **SD** | **Mean** | **SD** |
| **IDE [°]** | **1** | 2.12 | 5.54 | 2.01 | 5.78 | **1** | -22.04 | 8.69 | -18.12 | 10.11 | **1** | 37.42 | 12.62 | 49.13 | 14.72 |
|  | **2** | 2.54 | 4.08 | 0.87 | 6.01 | **2** | -19.12 | 14.61 | -13.35 | 7.28 | **2** | 35.27 | 13.97 | 48.41 | 17.37 |
|  | **3** | 0.83 | 3.58 | -0.00 | 5.39 | **3** | -13.74 | 10.40 | -8.86 | 5.73 | **3** | 35.41 | 13.86 | 45.77 | 14.84 |
|  | **4** | 0.96 | 5.19 | 1.41 | 4.54 |  |  |  |  |  |  |  |  |  |  |
| **PL [mm]** | **1** | 77.78 | 1.78 | 78.42 | 2.64 | **1** | 86.52 | 8.44 | 89.43 | 7.78 | **1** | 94.49 | 12.64 | 108.17 | 21.08 |
|  | **2** | 77.17 | 1.82 | 77.56 | 2.80 | **2** | 81.95 | 6.27 | 83.93 | 5.80 | **2** | 90.48 | 12.45 | 102.54 | 21.31 |
|  | **3** | 77.05 | 3.10 | 78.18 | 1.98 | **3** | 80.14 | 5.97 | 81.24 | 4.54 | **3** | 90.66 | 12.60 | 100.06 | 20.74 |
|  | **4** | 76.86 | 2.42 | 77.93 | 2.20 |  |  |  |  |  |  |  |  |  |  |
| *RT [ms]* | *1* | *508.12* | *103.23* | *430.42* | *98.02* | *1* | *497.67* | *173.43* | *431.57* | *111.12* | *1* | *489.99* | *151.05* | *456.97* | *115.72* |
|  | *2* | *463.13* | *89.83* | *433.55* | *87.50* | *2* | *471.84* | *139.82* | *430.65* | *93.51* | *2* | *499.06* | *152.14* | *441.29* | *94.31* |
|  | *3* | *453.28* | *86.67* | *431.54* | *85.20* | *3* | *453.67* | *114.83* | *426.03* | *74.55* | *3* | *474.54* | *143.12* | *438.47* | *103.51* |
|  | *4* | *450.51* | *94.16* | *427.85* | *87.96* |  |  |  |  |  |  |  |  |  |  |
| *MT [ms]* | *1* | *1772.7* | *575.8* | *1589.2* | *467.0* | *1* | *1794.3* | *544.0* | *1585.2* | *328.0* | *1* | *1752.0* | *427.9* | *1644.6* | *343.6* |
|  | *2* | *1559.8* | *483.0* | *1470.6* | *361.3* | *2* | *1498.2* | *459.3* | *1400.2* | *290.9* | *2* | *1705.6* | *478.6* | *1561.6* | *359.8* |
|  | *3* | *1496.4* | *499.2* | *1417.9* | *336.8* | *3* | *1387.6* | *408.5* | *1327.2* | *268.4* | *3* | *1674.5* | *436.8* | *1530.2* | *368.3* |
|  | *4* | *1466.1* | *447.6* | *1412.0* | *359.5* |  |  |  |  |  |  |  |  |  |  |
| *AE [mm]* | *1* | *2.234* | *0.916* | *1.960* | *0.914* | *1* | *3.016* | *1.379* | *2.542* | *0.905* | *1* | *2.832* | *0.517* | *2.704* | *0.392* |
|  | *2* | *2.373* | *0.968* | *2.481* | *1.402* | *2* | *2.427* | *0.955* | *2.260* | *0.972* | *2* | *2.672* | *0.346* | *2.798* | *0.811* |
|  | *3* | *2.714* | *2.977* | *1.934* | *0.731* | *3* | *2.450* | *0.914* | *2.151* | *0.839* | *3* | *2.653* | *0.352* | *2.672* | *0.317* |
|  | *4* | *2.324* | *1.379* | *2.156* | *1.196* |  |  |  |  |  |  |  |  |  |  |
| **Adaptation Phase** | | | | | |  |  |  |  |  |  |  |  |  |  |
| **Variable** | **Session** | **NbOxTr** | | **AirTr** |  | **Session** | **NbOxTr** | | **AirTr** | | **Session** | **NbOxTr** | | **AirTr** | |
|  |  | **Mean** | **SD** | **Mean** | **SD** |  | **Mean** | **SD** | **Mean** | **SD** |  | **Mean** | **SD** | **Mean** | **SD** |
| **IDE [°]** | **1** | 56.99 | 12.79 | 57.50 | 9.61 | **8** | 34.87 | 11.87 | 47.37 | 16.71 | **15** | 30.22 | 12.85 | 41.62 | 13.15 |
|  | **2** | 48.06 | 19.12 | 57.72 | 13.52 | **9** | 34.63 | 13.45 | 48.86 | 15.03 | **16** | 31.66 | 11.00 | 44.44 | 14.95 |
|  | **3** | 42.02 | 14.39 | 52.87 | 15.00 | **10** | 35.24 | 13.14 | 49.43 | 18.07 | **17** | 31.28 | 15.12 | 44.42 | 16.21 |
|  | **4** | 38.61 | 11.48 | 52.74 | 16.70 | **11** | 32.35 | 12.10 | 50.70 | 15.00 | **18** | 30.32 | 11.69 | 44.89 | 16.95 |
|  | **5** | 42.26 | 18.82 | 51.41 | 17.42 | **12** | 31.42 | 14.97 | 44.82 | 15.37 | **19** | 31.37 | 14.32 | 43.72 | 15.60 |
|  | **6** | 38.38 | 14.98 | 54.52 | 16.49 | **13** | 31.96 | 13.24 | 45.64 | 13.87 | **20** | 30.49 | 13.17 | 41.18 | 14.91 |
|  | **7** | 36.77 | 17.82 | 53.02 | 15.35 | **14** | 29.04 | 11.54 | 44.48 | 16.49 |  |  |  |  |  |
| **PL [mm]** | **1** | 119.75 | 32.13 | 147.78 | 33.42 | **8** | 91.38 | 11.90 | 102.35 | 21.45 | **15** | 87.68 | 11.19 | 96.73 | 17.51 |
|  | **2** | 103.90 | 16.62 | 117.31 | 22.46 | **9** | 90.50 | 12.00 | 100.42 | 21.26 | **16** | 86.83 | 10.00 | 96.45 | 17.95 |
|  | **3** | 102.65 | 19.57 | 113.12 | 20.48 | **10** | 90.04 | 10.89 | 100.05 | 20.58 | **17** | 86.69 | 9.47 | 98.66 | 20.53 |
|  | **4** | 102.93 | 21.08 | 111.37 | 19.58 | **11** | 91.26 | 16.51 | 99.93 | 19.67 | **18** | 86.20 | 6.85 | 97.16 | 18.02 |
|  | **5** | 100.60 | 20.91 | 112.07 | 27.45 | **12** | 90.06 | 12.43 | 99.29 | 21.05 | **19** | 86.99 | 11.40 | 96.02 | 17.53 |
|  | **6** | 95.51 | 14.42 | 107.31 | 19.48 | **13** | 88.79 | 11.23 | 100.41 | 25.12 | **20** | 86.90 | 10.31 | 95.82 | 16.85 |
|  | **7** | 92.33 | 12.25 | 105.75 | 22.14 | **14** | 87.90 | 10.91 | 99.42 | 21.44 |  |  |  |  |  |
| *RT [ms]* | *1* | *631.98* | *303.80* | *562.83* | *243.61* | *8* | *502.29* | *152.57* | *464.92* | *133.37* | *15* | *471.62* | *145.16* | *439.08* | *114.20* |
|  | *2* | *617.91* | *290.01* | *505.03* | *178.55* | *9* | *513.75* | *200.99* | *449.12* | *126.19* | *16* | *468.90* | *137.63* | *432.08* | *103.68* |
|  | *3* | *554.17* | *214.50* | *497.76* | *179.42* | *10* | *495.17* | *134.22* | *438.19* | *120.96* | *17* | *478.37* | *134.57* | *435.28* | *107.08* |
|  | *4* | *522.63* | *192.94* | *485.76* | *176.76* | *11* | *490.16* | *204.04* | *455.15* | *120.78* | *18* | *492.47* | *166.45* | *433.74* | *119.26* |
|  | *5* | *514.56* | *181.87* | *474.30* | *147.79* | *12* | *501.41* | *180.03* | *453.45* | *116.71* | *19* | *480.25* | *158.98* | *428.47* | *102.02* |
|  | *6* | *521.94* | *196.06* | *470.74* | *148.32* | *13* | *480.29* | *148.02* | *450.00* | *117.53* | *20* | *466.45* | *134.05* | *437.74* | *102.76* |
|  | *7* | *511.14* | *176.40* | *475.31* | *139.95* | *14* | *471.74* | *144.34* | *448.01* | *113.77* |  |  |  |  |  |
| *MT [ms]* | *1* | *3442.8* | *684.9* | *3598.9* | *999.1* | *8* | *1820.3* | *548.4* | *1834.9* | *493.4* | *15* | *1623.4* | *474.8* | *1631.1* | *426.8* |
|  | *2* | *2567.2* | *599.2* | *2539.3* | *667.8* | *9* | *1750.4* | *558.8* | *1726.7* | *461.0* | *16* | *1595.8* | *418.0* | *1619.6* | *406.5* |
|  | *3* | *2283.8* | *520.2* | *2256.8* | *563.4* | *10* | *1728.2* | *473.4* | *1704.3* | *430.0* | *17* | *1578.9* | *428.4* | *1584.9* | *411.6* |
|  | *4* | *2242.3* | *543.4* | *2116.0* | *533.5* | *11* | *1694.8* | *442.1* | *1726.3* | *424.9* | *18* | *1585.4* | *450.7* | *1554.8* | *364.4* |
|  | *5* | *2014.8* | *494.8* | *2109.7* | *600.1* | *12* | *1665.3* | *467.1* | *1685.5* | *398.4* | *19* | *1566.2* | *460.2* | *1549.2* | *437.4* |
|  | *6* | *1964.8* | *491.6* | *1950.7* | *499.4* | *13* | *1670.0* | *452.1* | *1675.7* | *368.9* | *20* | *1560.2* | *435.4* | *1497.2* | *345.0* |
|  | *7* | *1896.0* | *516.9* | *1874.2* | *454.7* | *14* | *1638.4* | *474.9* | *1673.8* | *388.0* |  |  |  |  |  |
| *AE [mm]* | *1* | *2.934* | *0.722* | *2.879* | *0.607* | *8* | *2.700* | *0.481* | *2.640* | *0.325* | *15* | *2.574* | *0.283* | *2.537* | *0.234* |
|  | *2* | *2.739* | *0.560* | *2.535* | *0.234* | *9* | *2.817* | *0.625* | *2.625* | *0.372* | *16* | *2.644* | *0.346* | *2.549* | *0.272* |
|  | *3* | *2.705* | *0.367* | *2.732* | *0.585* | *10* | *2.588* | *0.254* | *2.733* | *0.621* | *17* | *2.661* | *0.340* | *2.572* | *0.295* |
|  | *4* | *2.592* | *0.262* | *2.802* | *1.132* | *11* | *2.654* | *0.331* | *2.589* | *0.346* | *18* | *2.615* | *0.325* | *2.601* | *0.462* |
|  | *5* | *2.638* | *0.301* | *2.596* | *0.318* | *12* | *2.684* | *0.337* | *2.577* | *0.302* | *19* | *2.687* | *0.557* | *2.559* | *0.237* |
|  | *6* | *2.695* | *0.330* | *2.612* | *0.309* | *13* | *2.580* | *0.250* | *2.521* | *0.262* | *20* | *2.681* | *0.365* | *2.650* | *0.357* |
|  | *7* | *2.655* | *0.306* | *2.576* | *0.278* | *14* | *2.619* | *0.330* | *2.573* | *0.419* |  |  |  |  |  |
