# Supplementary material for: Boost your brain: a simple 100% normobaric oxygen treatment improves human motor learning processes
Source: Front Neurosci. 2023 Jul 11;17:1175649. doi: 10.3389/fnins.2023.1175649 (PMC10366362; doi:10.3389/fnins.2023.1175649)
Supplement: Supplementary file 2 [file Table_2.DOCX]

**Suppl. File II:** Visuomotor adaptation task statistics of the repeated-measures mixed ANOVA’s for Baseline, Adaptation, After-Effect, and Refresher phases for Group (NbOxTr, AirTr) and Session (Baseline 1-4; Adaptation 1-20; After-Effect & Refresher 1-3) and Group x Session for all dependent variables (IDE, PL, RT, MT, AE) of the. Note, gas treatment was only provided during the Adaptation phase. Abbreviations: NbOxTr = 100% normobaric oxygen treatment, AirTr = medical air treatment; IDE = Initial Direction Error, PL = Path length, RT = Reaction time, MT = Movement time, AE = Absolute endpoint error; ƞ2 = partial eta square.

| **Baseline Phase** | | | | | | | | | |
| --- | --- | --- | --- | --- | --- | --- | --- | --- | --- |
| **Variable** | **Group (NbOxTr, AirTr)** | | | **Session (1, 2, 3, 4)** | | | **Group*Session** | | |
|  | **F (1, 38)** | **p-value** | **ƞ^2^** | **F (3, 114)** | **p-value** | **ƞ^2^** | **F (3, 114)** | **p-value** | **ƞ^2^** |
| **IDE** | 0.61634 | 0.434279 | 0.015961 | 0.73511 | 0.533170 | 0.015961 | 0.30130 | 0.824398 | 0.007868 |
| **PL** | 1.57 | 0.217933 | 0.039665 | 2.03 | 0.113393 | 0.050753 | 0.55 | 0.647067 | 0.014347 |
| *RT* | *1.951* | *0.170533* | *0.048842* | *5.221* | *0.002053* | *0.120801* | *5.108* | *0.002365* | *0.118484* |
| *MT* | *0.5707* | *0.454650* | *0.014795* | *17.4663* | *0.000000* | *0.314899* | *1.1586* | *0.328804* | *0.029586* |
| *AE* | *0.7018* | *0.407428* | *0.018133* | *0.5507* | *0.648708* | *0.014284* | *0.9761* | *0.406688* | *0.025043* |
| **Adaptation Phase** | | | | | | | | | |
| **Variable** | **Group (NbOxTr, AirTr)** | | | **Session (1, 2, …, 18, 19, 20)** | | | **Group*Session** | | |
|  | **F (1, 38)** | **p-value** | **ƞ^2^** | **F (19, 722)** | **p-value** | **ƞ^2^** | **F (19, 722)** | **p-value** | **ƞ^2^** |
| **IDE** | **10.4375** | **0.002550** | **0.215484** | **20.0502** | **0.00000** | **0.345394** | **1.9841** | **0.007543** | **0.049621** |
| **PL** | **4.798** | **0.034698** | **0.112109** | **48.693** | **0.00000** | **0.561670** | **2.054** | **0.005200** | **0.051278** |
| *RT* | *1.0605* | *0.309612* | *0.027150* | *9.6457* | *0.00000* | *0.202446* | *0.6512* | *0.867271* | *0.016848* |
| *MT* | *0.0001* | *0.994188* | *0.000001* | *154.5430* | *0.00000* | *0.802642* | *0.5664* | *0.930133* | *0.014687* |
| *AE* | *0.444* | *0.509430* | *0.011538* | *0.232* | *0.023863* | *0.044209* | *0.688* | *0.932785* | *0.017790* |
| **After-Effect Phase** | | | | | | | | | |
| **Variable** | **Group (NbOxTr, AirTr)** | | | **Session (1, 2, 3)** | | | **Group*Session** | | |
|  | **F (1, 38)** | **p-value** | **ƞ^2^** | **F (2, 76)** | **p-value** | **ƞ^2^** | **F (2, 76)** | **p-value** | **ƞ^2^** |
| **IDE** | **4.9608** | **0.031928** | **0.115473** | **10.3396** | **0.000107** | **0.213896** | 0.1145 | 0.891995 | 0.003003 |
| **PL** | 1.014 | 0.320368 | 0.025985 | **68.367** | **0.000000** | **0.642748** | 0.998 | 0.373402 | 0.025991 |
| *RT* | *1.4636* | *0.233826* | *0.037088* | *3.7675* | *0.027538* | *0.090201* | *2.3325* | *0.103965* | *0.057831* |
| *MT* | *1.0097* | *0.321327* | *0.025884* | *101.4274* | *0.000000* | *0.727457* | *5.1449* | *0.008025* | *0.119248* |
| *AE* | *1.0867* | *0.303799* | *0.027801* | *15.8257* | *0.000002* | *0.294017* | *1.3441* | *0.266896* | *0.034163* |
| **Refresher Phase** | | | | | | | | | |
| **Variable** | **Group (NbOxTr, AirTr)** | | | **Session (1, 2, 3)** | | | **Group*Session** | | |
|  | **F (1, 38)** | **p-value** | **ƞ^2^** | **F (2, 76)** | **p-value** | **ƞ^2^** | **F (2, 76)** | **p-value** | **ƞ^2^** |
| **IDE** | **6.9985** | **0.011799** | **0.1555528** | **2.7754** | **0.068654** | **0.068654** | 0.7493 | 0.476159 | 0.019337 |
| **PL** | **4.603** | **0.038375** | **0.108037** | **40.695** | **0.000000** | **0.517125** | **4.720** | **0.011692** | **0.110482** |
| *RT* | *1.1257* | *0.295382* | *0.028773* | *3.1357* | *0.049141* | *0.076229* | *1.7626* | *0.178539* | *0.044328* |
| *MT* | *1.0951* | *0.301953* | *0.028011* | *11.5214* | *0.000043* | *0.232655* | *0.5403* | *0.584768* | *0.014020* |
| *AE* | *0.002* | *0.966962* | *0.000046* | *0.937* | *0.396435* | *0.024054* | *1.291* | *0.281085* | *0.032846* |
