# Supplementary material for: Boost your brain: a simple 100% normobaric oxygen treatment improves human motor learning processes
Source: Front Neurosci. 2023 Jul 11;17:1175649. doi: 10.3389/fnins.2023.1175649 (PMC10366362; doi:10.3389/fnins.2023.1175649)
Supplement: Supplementary file 3 [file Table_3.DOCX]

**Suppl. file III.1:** Blood oxygenation mean descriptive results of the T-Test for groups (NbOxTr, AirTr) during Baseline, Adaptation, and After-Effect & Refresher phases. Note, gas treatment was only provided during the Adaptation phase. Abbreviations: NbOxTr = 100% normobaric oxygen treatment, AirTr = medical air treatment

| **Phase** | **NbOxTr** | | | **AirTr** | | |
| --- | --- | --- | --- | --- | --- | --- |
|  | **Mean** | | **SD** | | **Mean** | **SD** |
| **Baseline** | 98.3 | 0.73270 | | | 98.45 | 0.82558 |
| **Adaptation** | 98.95 | 0.22361 | | | 98.5 | 0.76089 |
| **After-Effect & Refresher** | 98.2 | 0.83351 | | | 98.25 | 0.71635 |

**Suppl. File III.2:** Blood oxygenation statistical outcomes of the T-Tests comparing groups (NbOxTr, AirTr) during Baseline, Adaptation, and After-Effect & Refresher phases. Note, gas treatment was only provided during the Adaptation phase. T-tests were performed assuming equal variance for Baseline, After-effect and Refresher Phase and assuming unequal variance for Adaptation phase.

| **Baseline Phase** | | **Adaptation Phase** | | | **After-effect & Refresher Phase** | |
| --- | --- | --- | --- | --- | --- | --- |
| **T (38)** | **p-value** | **T (22)** | **p-value** | **T (38)** | | **p-value** |
| 0.607726 | 0.546984 | **-2.537584** | **0.015386** | 0.203456 | | 0.419947 |

**Suppl. file III.3:** Room air oxygen percentage mean descriptive results of the T-Test for groups (NbOxTr, AirTr) during Baseline, Adaptation, and After-Effect & Refresher phases. Note, gas treatment was only provided during the Adaptation phase. Abbreviations: NbOxTr = 100% normobaric oxygen treatment, AirTr = medical air treatment

| **Phase** | **NbOxTr** | | | **AirTr** | | |
| --- | --- | --- | --- | --- | --- | --- |
|  | **Mean** | | **SD** | | **Mean** | **SD** |
| **Baseline** | 20.61 | 0.0999 | | | 20.58 | 0.1105 |
| **Adaptation** | 20.61 | 0.0852 | | | 20.58 | 0.0951 |
| **After-Effect & Refresher** | 20.62 | 0.1005 | | | 20.575 | 0.0966 |

**Suppl. File III.4:** Room air oxygen percentage statistical outcomes of the T-Tests comparing groups (NbOxTr, AirTr) during Baseline, Adaptation, and After-Effect & Refresher phases. Note, gas treatment was only provided during the Adaptation phase. T-tests were performed assuming equal variance for Baseline, After-effect and Refresher Phase and assuming unequal variance for Adaptation phase.

| **Baseline Phase** | | **Adaptation Phase** | | | **After-effect & Refresher Phase** | |
| --- | --- | --- | --- | --- | --- | --- |
| **T (38)** | **p-value** | **T (22)** | **p-value** | **T (38)** | | **p-value** |
| -0.8649 | 0.3979 | -1.3708 | 0.1864 | -1.5282 | | 0.1429 |
